# Assessment of antibiotic treatment on *Anopheles darlingi* survival and susceptibility to *Plasmodium vivax*

**DOI:** 10.3389/fmicb.2022.971083

**Published:** 2022-10-05

**Authors:** Najara Akira Costa dos Santos, Felipe Neves Magi, Alice Oliveira Andrade, Alessandra da Silva Bastos, Soraya dos Santos Pereira, Jansen Fernandes Medeiros, Maisa da Silva Araujo

**Affiliations:** ^1^Postgraduate Program in Experimental Biology, Federal University of Rondonia/Fiocruz Rondonia, Porto Velho, Brazil; ^2^Platform of Production and Infection of Malaria Vectors (PIVEM), Laboratory of Entomology, Fiocruz Rondonia, Porto Velho, Brazil; ^3^Antibody Engineering Laboratory, Fiocruz Rondonia, Porto Velho, Brazil

**Keywords:** Microbiota, *Plasmodium vivax*, *Anopheles darlingi*, antibiotic, artificial infection, vectorial capacity, experimental models

## Abstract

Antibiotic treatment has been used to enhance anopheline susceptibility to *Plasmodium* infection, because bacterial microbiota play a fundamental role in modulating the vector competence of mosquitoes that transmit *Plasmodium* parasites. However, few studies have examined the impact of antibiotic treatments on *Plasmodium vivax* sporogonic development in neotropical anopheline mosquitoes. Herein, we assessed the impact of antibiotic treatment on *P. vivax* development and survival in *Anopheles darlingi*, the main vector of malaria in the Amazon region. Female mosquitoes were treated continuously with antibiotics to impact the gut bacterial load and then tested for prevalence, infection intensity, and survival in comparison with untreated mosquitoes. Antibiotic-fed mosquitoes had not dramatic impact on *P. vivax* development previously observed in *P. falciparum*. However, antibiotic treatment increases mosquito survival, which is known to increase vectorial capacity. These findings raise questions about the effect of antibiotics on *P. vivax* development and survival in *An. darlingi*.

## Introduction

*Anopheles darlingi* (Root) is the main malaria vector in the Amazon region, and it occurs mainly in deforested areas (Vittor et al., 2006). This vector is highly susceptible to *Plasmodium vivax* and *Plasmodium falciparum* parasites and exhibits accentuated anthropophilic behavior, which ensures malaria transmission even when vector density is low (Tadei and Dutary-Thatcher, 2000; Hiwat and Bretas, 2011).

Anopheline mosquitoes become infected with *Plasmodium* spp. by feeding on the blood of a vertebrate host infected with gametocytes. In the mosquito’s midgut, modification in temperature and pH, and the presence of xanthurenic acid cause gametocytes to divide into macro and microgametes *via* gametogenesis, and fertilization results in the formation of a zygote. The zygote transforms into a mobile structure known as an ookinete. Approximately 24 h after the infectious blood meal, the ookinete traverses the peritrophic matrix and midgut epithelium. Ookinetes then invade the mosquito’s intestinal wall and form oocysts. After 8–10 days of infection, the oocysts rupture and release thousands of sporozoites into the mosquito hemocoel. The sporozoites invade the salivary glands and are transferred *via* blood meal to the next vertebrate host, thereby perpetuating the transmission cycle (Vaughan et al., 1992; Zollner et al., 2006).

Although, *Plasmodium* spp. susceptibility among anopheline mosquitoes seems to be conditioned by genetic control, other factors may act in combination to mediate vector-parasite interaction (Vernick et al., 2005). In this context, the midgut microbiota plays a key role in regulating mosquito vector competence for *Plasmodium* transmission. The microbiota is composed of bacteria, viruses, and yeast (Ricci et al., 2011; Wang et al., 2011; Chandler et al., 2015; Bozic et al., 2017). Most studies to date have examined the role and diversity of bacteria in the mosquito midgut (Dong et al., 2009; Meister et al., 2009). Microbiota-mosquito interaction initiates two different responses to control the pathogen: first, an indirect induction of the immune response is mediated by the microbiota (Gendrin and Christophides, 2013); second, the direct effect of some bacteria metabolites affect the *Plasmodium* midgut stages, as observed in *Enterobacter* ESP_Z and *Chromobacterium* in *Anopheles gambiae* (Cirimotich et al., 2011; Ramirez et al., 2014). In addition, the microbiota may also inhibit *Plasmodium* growth *via* metabolic competition inside the midgut (Chabanol et al., 2020).

The bacterial microbiota plays a beneficial role in the normal development of *An. gambiae, Aedes aegypti* and *Georgecraigius atropalpus* larvae (Chouaia et al., 2012; Coon et al., 2014). In *Anopheles coluzzi*, bacteria in the midgut is involved in peritrophic matrix formation after blood feeding (Rodgers et al., 2017), and antibiotic treatment assays have shown that *Ae. aegypti* bacterial microbiota contribute to blood digestion and consequently improve egg production (Gaio et al., 2011). However, intestinal microbiota has been shown to have a detrimental effect on survival and fertility in adult *An. gambiae* mosquitoes relative to mosquitoes treated with antibiotics (Gendrin et al., 2015).

The impact of bacterial microbiota on the vector competence of anopheline mosquitoes has also been observed in antibiotic treatment assays. Studies of *An. gambiae*, *An. coluzzi* and *Anopheles stephensi* have shown that using antibiotics to impact bacterial load before infected blood meals leads to a significant increase in *P. falciparum*, *Plasmodium berghei*, *Plasmodium vinckei petteri* and *Plasmodium gallinaceum* infection relative to untreated mosquitoes (Pumpuni et al., 1993; Beier et al., 1994; Dong et al., 2009; Sharma et al., 2013; Gendrin et al., 2015, 2016; Martinez-de la Puente et al., 2021).

In contrast, limited information exists on *P. vivax* interaction with Neotropical anopheline mosquitoes and the bacteria of their microbiota (Gonzalez-Ceron et al., 2003; Moreno et al., 2018)*. Plasmodium vivax* is the most widely distributed species of *Plasmodium* in the world and the main parasite in the Amazon region. Moreover, because *P. vivax* can remain latent in the liver, recovered carriers may relapse and thus maintain the malaria transmission cycle even in the face of control and elimination strategies (Rougeron et al., 2020). Because *P. vivax* is so virulent, new strategies for vivax malaria prevention have been developed to block malaria transmission in endemic areas (Vallejo et al., 2016). However, control of malaria transmission in the Amazon will require specific, vector-focused approaches based on a thorough investigation of Neotropical anopheline biology (Rocha et al., 2020) and the complex interactions of *P. vivax* with its primary mosquito vector, which, in Brazil, is *An. darlingi*. Recently, well-established laboratory colonies of *An. darlingi* have become available, thereby allowing targeted investigations of *P. vivax*-vector interaction (Moreno et al., 2014; Villarreal-Treviño et al., 2015; Araujo et al., 2019; Puchot et al., 2022), and providing a basis for *P. vivax*–*An. darlingi* modeling that can be used to develop new transmission-blocking strategies in endemic areas.

To establish a consistent *P. vivax*–*An. darlingi* model, mosquito colony susceptibility to *P. vivax* needs to be increased using direct membrane feeding assay (DMFA), and the impact of antibiotic treatment on *P. vivax*–*An. darlingi* interaction needs to be assessed. We used *P. vivax* samples and a well-established colony of *An. darlingi* to assay the effects of continuous antibiotic treatment on the *P. vivax* sporogonic cycle, and to determine the impact of this treatment on the survival of infected versus uninfected mosquitoes.

## Materials and methods

### Ethics statement

All experiments were performed with the approval of the Ethics Committee at the Centro de Pesquisa em Medicina Tropical (CEPEM) (n° 530,106). All patients at the CEPEM diagnosed with *P. vivax* malaria by microscopy (≥1 gametocyte by 200 leukocytes), who were ≥18 years of age, not pregnant, not indigenous, and absent severe or complicated malaria were invited to participate in the study. Informed consent was read and signed by each volunteer.

### Mosquito rearing

Female mosquitoes were obtained from the *An. darlingi* colony of the Platform for Production and Infection of Malaria Vectors (PIVEM) FIOCRUZ-RO/Brazil, established and maintained since 2018 by Araujo et al. (2019). The *An. darlingi* colony was maintained on rabbit blood and adults were fed with 15% honey solution *ad libitum* at 26 ± 1°C and 70 ± 10% relative humidity, on a 12-h/12-h day-night cycle. Larvae were fed daily with TetraMin^®^ Marine fish food, and reared in 1 l of distillated water which was changed twice a week.

### Antibiotic treatment

**Figure 1:**
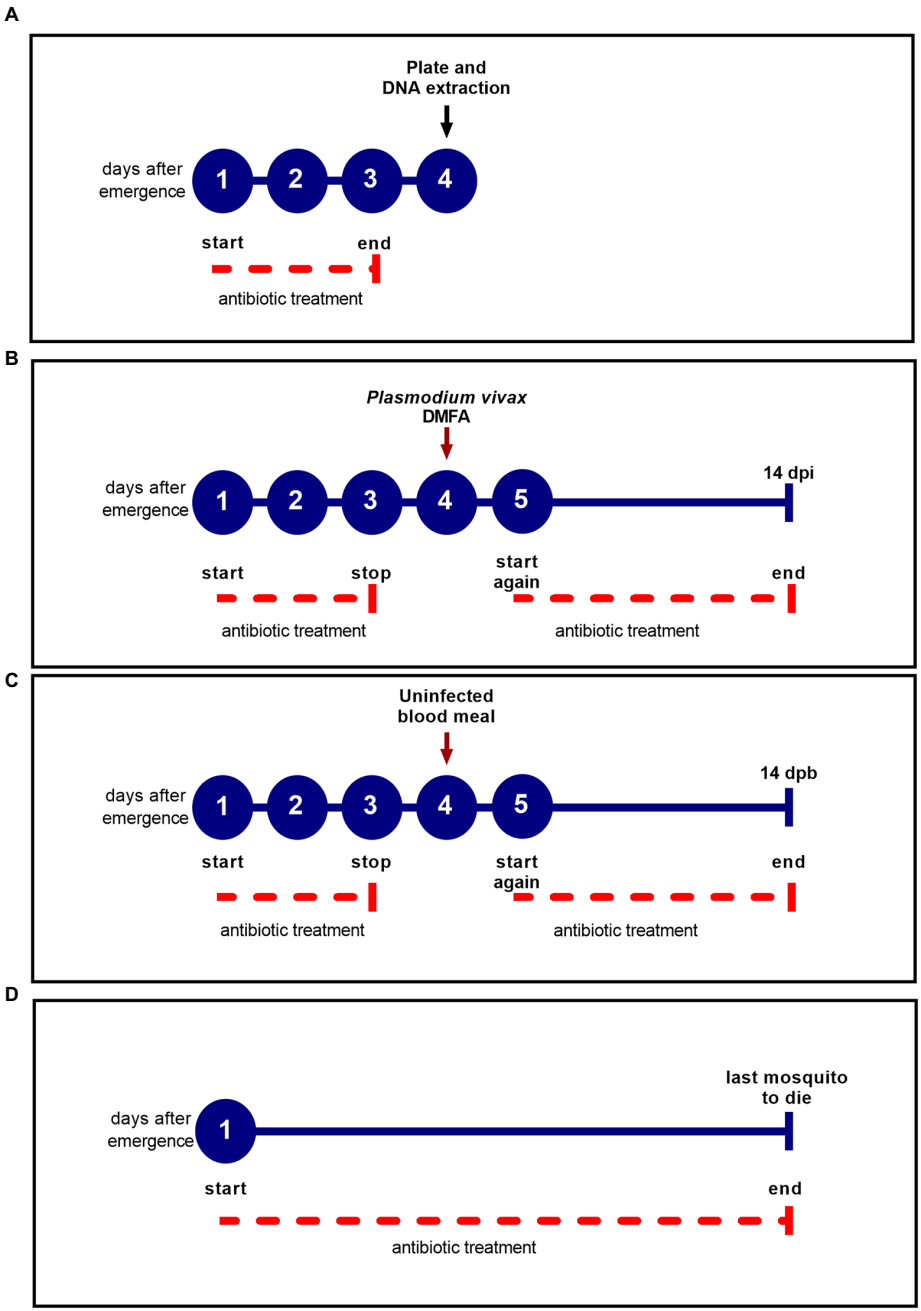
Antibiotic treatment scheme for each experiment. The blue circles represent the days after female emergence and the solid blue line represents the experiment duration. The dotted red line indicates the antibiotic treatment period. **(A)** Experimental design to determine the efficacy of the antibiotic treatment before the DMFA; **(B)** Experimental design for DMFA; **(C)** Experimental design for uninfected blood meal; **(D)** Experimental design for survival assay.

In order to assess whether antibiotic-treatment affects susceptibility and survival, emerged female mosquitoes were fed 15% honey solution mixed with antibiotics daily (penicillin–streptomycin 10 U/ml-μg/mL from GIBCO and gentamicin sulfate 15 μg/ml) (Touré et al., 2000; Dong et al., 2009) until the end of the experiments (Figure 1). The mixture was named PSG (penicillin–streptomycin + gentamicin), and the mosquito batches that received the antibiotic treatment were defined as treated groups. Untreated groups were maintained on a 15% honey solution in accordance with the *An. darlingi* colony protocol (Araujo et al., 2019). In general, penicillin–streptomycin and gentamicin generate synergistic antimicrobial activity. While penicillin, a wide spectrum antibiotic of the β-lactamic family, acts by inhibiting peptidoglycan synthesis of bacteria, streptomycin and gentamicin are aminoglycoside antibiotics that bind to the 30S ribosomal unit and irreversibly interfere with protein synthesis, and exhibit activity against Gram-negative and Gram-positive bacteria (Spratt, 1980; Vakulenko and Mobashery, 2003).

To determine the efficacy of the antibiotic treatment, the PSG solution was administered for three days following emergence and prior to blood feeding. We plated individually dissected midguts of treated and untreated groups in LB agar and incubated the plates for 48 h at 27°C, according to Dong et al. (2009) (Figure 1A). The choice of 4th day after emergence was consistent of the day which mosquitoes were submitted to blood feeding (Figures 1B,C). The dissections were performed with autoclaved materials, and 70% ethanol was used to wipe materials that could not be autoclaved. Mosquito surfaces were sterilized in 70% ethanol for 5 min, then rinsed in sterile 1× PBS solution (Boissière et al., 2012). Each midgut (five midguts per group) was ground in 100 μl of sterile PBS using sterile pestles, and a serial dilution (10-fold serial dilution) was performed to estimate the concentration of bacteria in each midgut. Twenty milliliters of the serial dilution (0.1) were plated in technical duplicate, and all experiments were performed twice at different time points. The colony forming unit (CFU) was determined by: (number of colonies) × (dilution factor)/plated volume (20 μl), according to Tchioffo et al. (2013), and expressed as CFU/midgut.

Likewise, qPCR was performed to assess the DNA bacterial load of female mosquitoes aged 4 days that had been treated with antibiotic for 3 days prior to blood feeding (Figure 1A). Treated and untreated mosquito groups were dissected as described above. DNA extraction was performed using DNeasy Blood and Tissue kit (Qiagen, Valencia, CA, USA), following the manufacturer’s protocol. A plasmid was constructed from the 180 bp amplified fragment of the 16S rRNA gene and then cloned into the pGEM-T Easy plasmid vector (Promega). The 180 bp fragment was obtained using primers UNI-F (5′ ACTCCTACGGGAGGCAGCAGT 3′) and UNI-R (5′ ATTACCGCGGCTGCTGGC 3′) (Hartman et al., 2009). Standard curves were constructed using serial dilution (10^7^ to 10^1^) of the UNI-16S plasmid. Each qPCR reaction contained 2 μl of DNA, 7 μl of SYBR Green PCR Master Mix 2X (Applied Biosystems™), 0.3 μM of each oligonucleotide primer (UNI-F 5′ ACTCCTACGGGAGGCAGCAGT 3′; UNI-R 5′ ATTACCGCGGCTGCTGGC 3′) and nuclease-free water for a total volume of 15 μl. Amplification conditions were: 50°C for 2 min; denaturation at 95°C for 10 min; 30 cycles at 95°C for 15 s; 65.5°C for 1 min, and a final melting curve analysis from 60°C to 95°C for 1 min. All reactions were performed in triplicate and a nontemplate control and serial dilution of plasmids were included in each run.

### Blood collection from *Plasmodium vivax* patients for DMFA

Blood samples from *P. vivax-*infected humans were collected by venipuncture using heparinized Vacutainer tubes (10 ml). The tubes were stored in a water flask at 37°C and transported to the PIVEM insectary for DMFA. Using a microscope (100×), asexual and sexual stage parasite densities were estimated for each volunteer by counting the number of parasites per 200 leukocytes in a thick blood smear stained with 10% Giemsa (WHO, 2010).

### Artificial infection experiment

Prior to DMFA, four-day old *An. darlingi* female mosquitoes from each group (treated with antibiotics and untreated) were deprived of the PSG or honey solution for 12 h (Figure 1B). Batches of 100 mosquitoes per group were fed on *P. vivax* blood isolates. Two milliliters of heparinized blood were offered to both groups *via* DMFA, and mosquitoes were allowed to feed for 30 min. Glass membrane feeders had a parafilm membrane and were connected to a water bath to maintain a constant temperature of 37°C. Only fully engorged mosquitoes were used in the experiment. The day after the DMFA, the PSG solution and 15% honey solution were offered again daily *ad libitum* until 14 days post infection (dpi) (Figure 1B).

### Mosquito survival

Some studies have shown that treating mosquitoes with antibiotics for three days prior to infection or adding antibiotics to infected blood (Dong et al., 2009; Gendrin et al., 2015; Moreno et al., 2018) can affect *Anopheles* survival. To test the effect on *An. darlingi* survival of offering PSG daily, two different experiments were performed: (i) treated and untread mosquitoes submitted to DMFA using *P. vivax*-infected blood were analyzed for survival in comparison to groups fed with uninfected blood (Figures 1B,C). The mosquitoes fed with infected blood were denominated Pv^+^ and mosquitoes fed with uninfected blood were denominated Pv^−^. PSG solution and 15% honey solution were offered to the mosquito groups as described in Figure 1B, and daily mortality was recorded until 14-day post-blood meal (dpb); (ii) for treated and untreated mosquitoes that were fed only honey, daily mortality was recorded until the last mosquito died (Figure 1D). Five replicates were performed with a total of approximately 1,500 female mosquitoes.

### Oocysts and sporozoites of infected mosquitoes

At 7 and 14 days post-infection (dpi), infected mosquitoes were dissected to count oocysts and sporozoites, respectively. Midguts were dissected in PBS 1X under stereomicroscope and stained with 0.2% mercurochrome (SIGMA) to examine oocyst presence and quantity under microscopy (10X).

To estimate the number of sporozoites, salivary glands were dissected, pooled (up to a maximum of five salivary glands per pool), ground with a glass pestle in 15 μl RPMI solution, and centrifuged at 6,000 rpm for 30 s; 10 μl was pipetted into a Neubauer chamber hemocytometer and sporozoites were counted under microscopy (40X).

### Statistical analysis

Exploratory analysis using summary statistics and graphs was performed to assess engorgement rate, prevalence, oocyst and sporozoite intensity, and survival rate. Engorgement rate and prevalence were analyzed by Chi-square test with Yates correction, and confidence intervals were calculated by Wald method. Oocyst and sporozoite intensities (number of oocyst and sporozoite per mosquito) were estimated for midguts and salivary glands having one or more parasites. A Mann–Whitney test was performed to check significant differences in intensity for oocysts and sporozoites, bacterial load (CFU/midgut) and DNA copy number/μl.

The Kaplan–Meier survival curve was used to estimate survival rates. Cox proportional hazards regression was employed to estimate the hazard ratio, and the likelihood-ratio test was performed to assess overall significance of the model. Statistical analyses were conducted in R program (version 3.6.3, R Foundation for Statistical Computing, Austria), and the graphs were constructed in GraphPad Prism (version 9.3.1).

## Results

### Antibiotic treatment effect on midgut bacterial load of *Anopheles darlingi*

**Figure 2:**
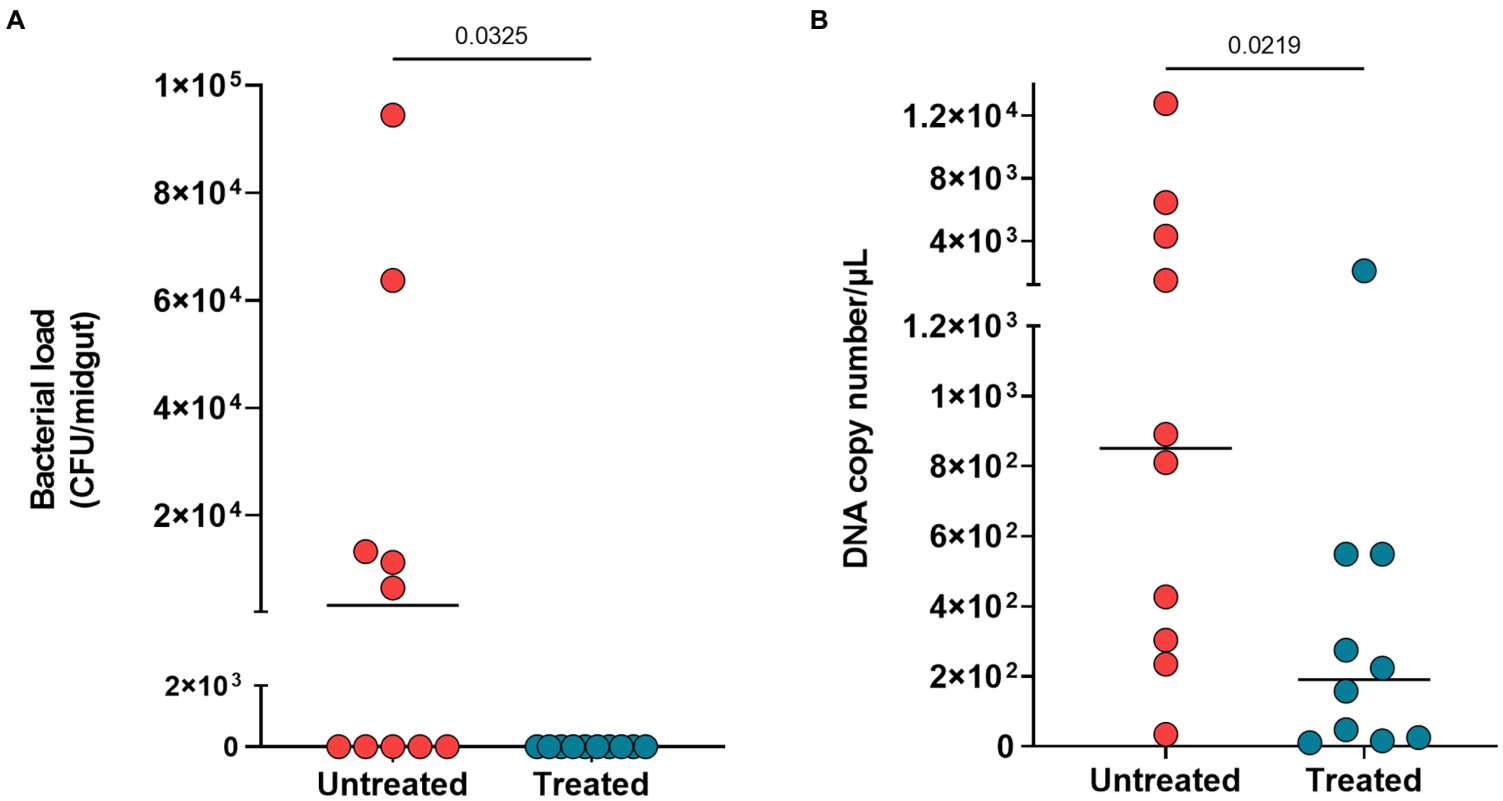
Midgut microbiota reduction after three days of antibiotic treatment. **(A)** Bacterial load of plated midguts untreated mosquitoes (red circles) and treated mosquitoes (blue circles). **(B)** DNA copy number per microliter of midgut from untreated and treated mosquitoes. Each circle represents midgut analyzed, and horizontal black bars represent the median value. The Mann Whitney test was used to determine significance for differences in reduction of bacterial load and DNA copy number.

The antibiotic treatment reduced the bacterial load of midgut microbiota as observed on LB plates (*p* = 0.032) (Figure 2A) and by DNA quantification of treated mosquitoes (*p* = 0.021) (Figure 2B).

### Effect of antibiotic treatment on *Plasmodium vivax* development in *Anopheles darlingi*

To assess the antibiotic-treatment effect on *An. darlingi* infected with *P. vivax,* we performed 10 DMFAs using 10 samples from *P. vivax* positive donors. However, one *P. vivax* positive sample failed to infect the control group (untreated mosquitoes) (see Supplementary Table S1), and this sample was excluded from the analysis.

Parasitemia and gametocytemia were estimated by microscopy of thick blood smear from *P. vivax* blood samples and ranged from 0 gametocyte/μL to 1,260 gametocyte/μL (Table 1). Data from each patient and each DMFA are shown in Supplementary Table S1.

**Table 1:** Results of direct membrane feeding assay for *Anopheles darlingi* treated and untreated with antibiotics.

Experimental group	Median gametocyte/μL (Min–Max)	Median assexual/μL (Min–Max)	Positive for oocyst/midgut dissected Prevalence (%)	*p-*value	Median oocyst (IQR)	*p-*value	Positive for sporozoite/mosquito dissected Prevalence (%)	*p-*value	Median sporozoites (IQR)	*p-*value
Treated Pv^+^	150 (0–1,260)	7,230 (180–13,590)	327/346 (94.5)	0.099	42 (12–109)	0.058	376/390 (96.4%)	0.004	6,480 (1,700 – 13,200)	0.155
Untreated Pv^+^			267/294 (90.8)		54 (21–116)		260/260 (100%)		7,267 (2,400–14,720)	

**Figure 3:**
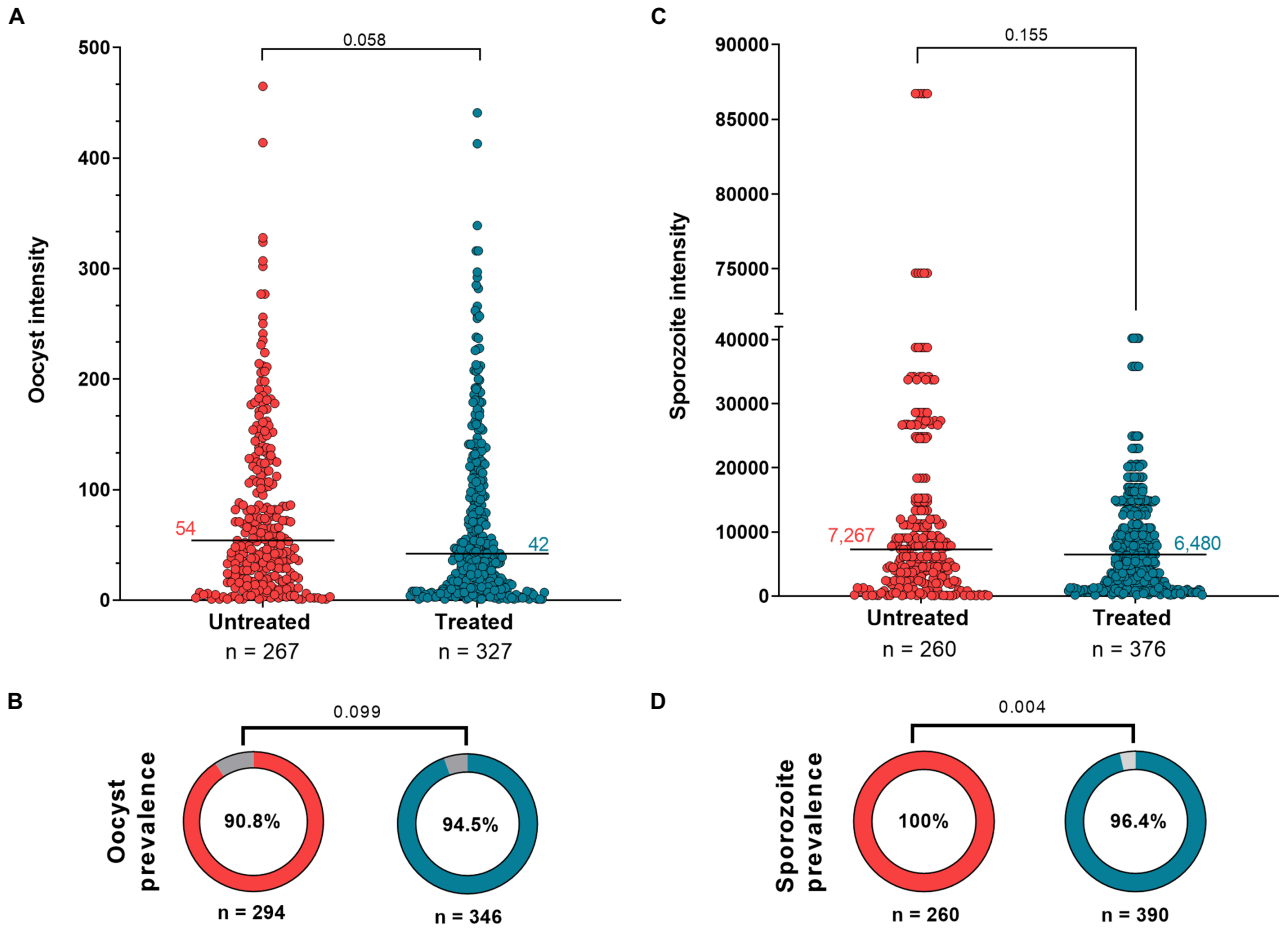
Antibiotic effect on *Anopheles darlingi* infected by *Plasmodium vivax*. **(A)** Oocyst intensity in untreated and treated mosquitoes; and **(C)** Sporozoite intensity in untreated and treated groups. The Mann Whitney test was used to compare groups. Each circle indicates a mosquito with at least 1 parasite. **(B,D)** show prevalence data of oocyst and sporozoite stages, respectively. The χ^2^ test was used to compare groups. The intensity and prevalence parameters were tested in nine independent DMFAs, which showed prevalence above 40% in the control group [the independent DMFA “5,033” was removed from analysis (Supplementary Table S1)]. Value of *p* less than 0.05 is considered statistically significant.

The effect of antibiotic treatment on the *P. vivax* development in *An. darlingi* is shown in Figure 3. Although, the antibiotic treatment did not have a significative impact on *An. darlingi* oocyst prevalence (*χ*^2^ = 2.718; z = 1.649; *p* = 0.099), we observed that the treated group exhibited a higher prevalence (94.5%; CI 95 91.5–96.5%) than the untreated group (90.8%; CI 95 86.9–93.6%) (Figure 3B; Table 1). Additionally, in most independent experiments we did not able to detect an increase in oocyst prevalence because the prevalence was 100% or close to 100% (see Supplementary Table S1).

No significant difference in infection intensity was observed between experimental groups (Mann–Whitney test, *U* = 39.719; *p* = 0.058). Oocyst intensity ranged from 1 to 441 (median 42; 95% CI 35–47; IQR 12–109) for the treated group, and 1–465 (median 54; 95% CI 44–65; IQR: 21–116) for the untreated group (Figure 3A; Table 1). In the treated group, sporozoite intensity was also less (median 6,480; 95% CI 5,120–7,520; IQR 1,700–13,200) than in the untreated group (median 7,267; CI 95% 5,280–8,240; IQR: 2,400–14,720), but the difference was not significant (Mann–Whitney test, *U* = 45,641; *p* = 0.155) (Figure 3C; Table 1). However, we observed a slight and significant reduction in sporozoite prevalence in the treated group (96.4; 95%CI 94–97.9%) compared to the untreated group (100%; 95% CI 98.2–100%) (*χ*^2^ = 2.813; *z* = 2.813; *p* = 0.004) (Figure 3D; Table 1). Data from each DMFA are shown in Supplementary Table S1.

### Antibiotic treatment effect on *Anopheles darlingi* survival

**Figure 4:**
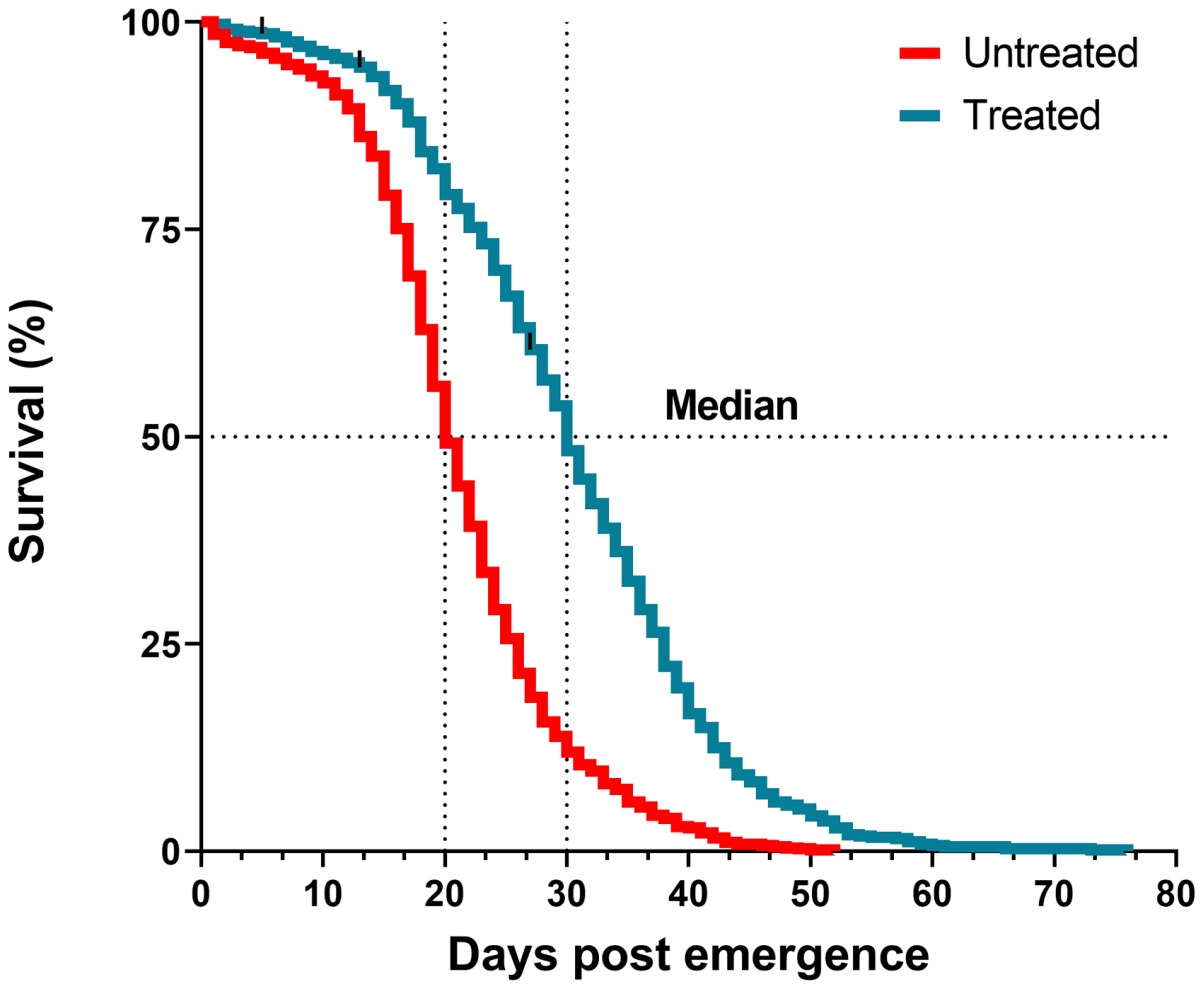
Survival curves of mosquitoes treated with antibiotics (blue line) and untreated (red line) until the last mosquito died. Log-rank test, *p* < 0.0001. Data are pooled from five independent experiments. Mosquitoes (*n* ~ 150) per experiment and per condition.

To assess the impact of the antibiotic-treatment on *An. darlingi* survival, we observed 714 mosquitoes from the treated group and 738 mosquitoes from the untreated group. The median survival time was 30 days for the treated group, and 20 days for the untreated group (Figure 4). The survival curve and a reduced hazard ratio (HR = 0.38; CI 95% 0.34–0.42; SE ± 0.5; *z* = −17.02; *p* < 0.0001) indicate that antibiotic treatment had a protective effect.

**Figure 5:**
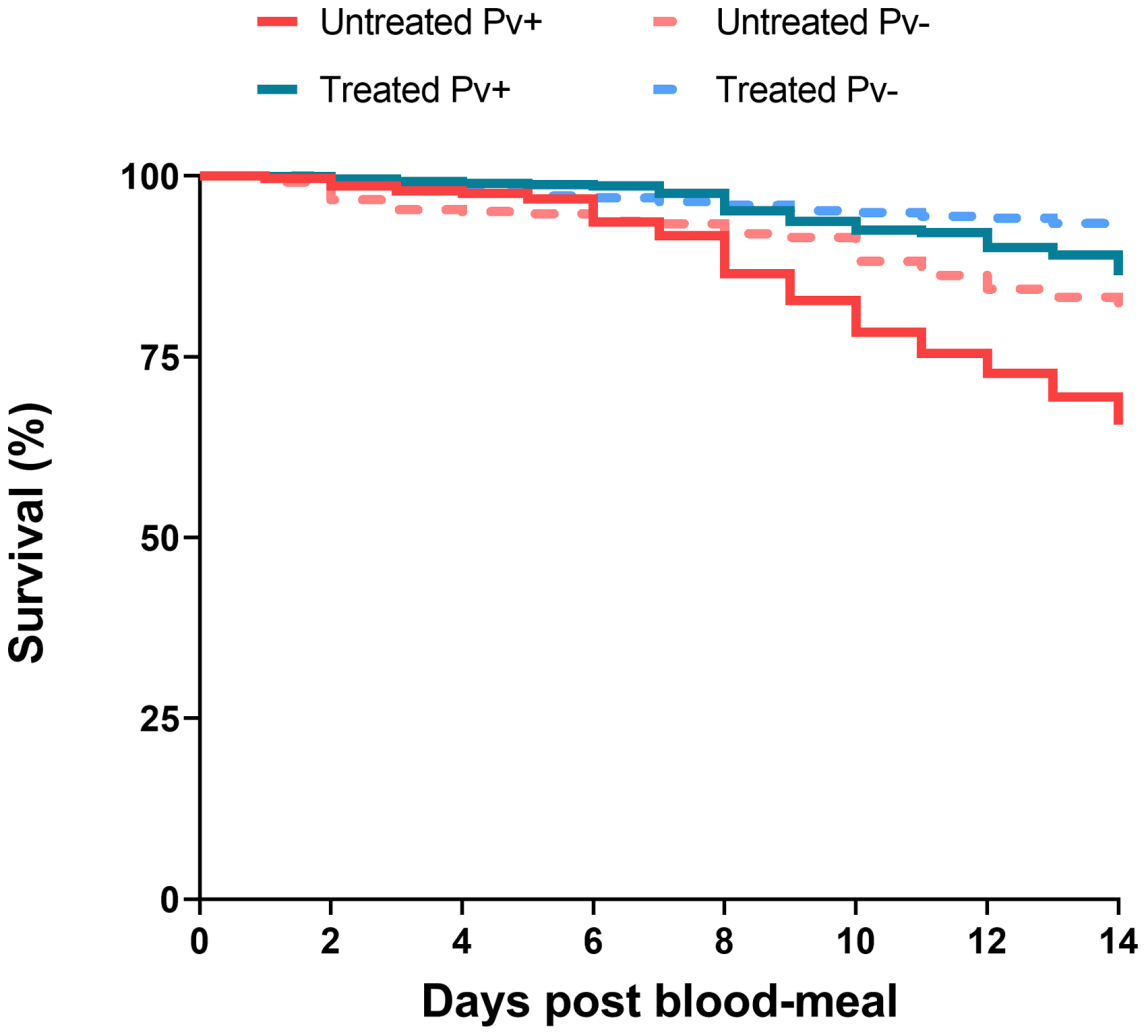
Survival curves of mosquitoes untreated and treated with antibiotics and fed on *Plasmodium vivax* positive samples (Pv^+^) or on uninfected blood samples (Pv^−^). Data are pooled from nine independent experiments with *P. vivax* positive groups (Pv^+^) and five independent experiments with uninfected groups (Pv^−^). Mosquitoes (*n* ~ 100) per experiment and per condition.

To assess the antibiotic-treatment effect on the survival of *An. darlingi* infected with *P. vivax*, mosquitoes fed with blood from five healthy donors were compared with mosquitoes fed on infected blood. A total of 1,537 infected mosquitoes and 759 uninfected mosquitoes were included in the survival analysis. In general, mosquito survival increased under antibiotic treatment, independent of whether mosquitoes were fed with infected or uninfected blood. The treated Pv^−^ group had the highest survival rate (92.2%; CI 95 89.5–94.8%), followed by the treated Pv^+^ group (86.1%; CI 95 83.1–89.3%), the untreated Pv^−^ group (80.2%; CI 95 76.2–84.4%) and the untreated Pv^+^ group (64.6%; CI 95 60.3–69.2%) (Figure 5).

The Cox models showed that antibiotic treatment had a protective effect on the mosquitoes until sporogonic development. The treated Pv^+^ group achieved an HR of 0.33 (CI 95% 0.25–0.43; SE ± 0.14; *z* = −7.83; *p* < 0.0001). This means that the daily risk of death for infected mosquitoes treated with antibiotics was 66.7% less than it was for the untreated Pv^+^ group. Daily risk of death was 36.4% less for the treated Pv^+^ group relative to the untreated Pv^−^ group (HR = 0.63; CI 95% 0.45–0.88; SE ± 0.16; *z* = −2.70; *p* = 0.0068), and 63% less for the treated Pv^−^ group relative to the untreated Pv^−^ group (HR = 0.37; CI 95% 0.24–0.56; SE ± 0.21; *z* = −4.61; *p* < 0.0001) (Table 2). In other words, regardless of infection, the antibiotic-treatment improved mosquito survival.

**Table 2:** Hazard ratio estimated by Cox model until 14 days post infection.

	Reference in the model			
	Untreated Pv^+^	Untreated Pv^−^	Treated Pv^−^	Treated Pv^+^
Untreated Pv^+^	–	1.91*	5.15*	3.00*
Untreated Pv^−^	0.52*	–	2.69*	1.57*
Treated Pv^−^	0.19*	0.37*	–	0.58*
Treated Pv^+^	0.33*	0.63*	1.71*	–

* *p <* 0.05.

We also assessed the impact of infection on survival as estimated by Cox model. The treated Pv^+^ group had an HR of 1.71 (CI 95% 1.12–2.61; SE ± 0.21; *z* = 2.50; *p* = 0.012) compared to the treated Pv^−^ group, and the untreated Pv^+^ group had an HR of 1.91 (CI 95% 1.44–2.52; SE ± 0.1; *z* = 4.58; *p* < 0.0001) compared to the untreated Pv^−^ group (Table 2). These values show an increased probability of death from infection; however, no differences were registered between groups until 7dpb (Supplementary Figure S1; Supplementary Table S2).

Treatment also exhibited a positive effect on engorgement rate. The treated Pv^+^ groups showed an increase of 11.4% in engorgement rate (89.9%; CI 95 87.7–91.7%) relative to the untreated Pv^+^ group (80.6%; IC 95 77.9–83.1%) (*χ*^2^ = 29.96; df = 1; *z* = 5.474; *p* < 0.0001). The same difference in engorgement rate was observed between treated Pv^−^ (79%; CI 95 75.2–82.3%) and untreated Pv^−^ mosquitoes (72.8%; CI 95 68.7–76.5%) (*χ*^2^ = 4.92; df = 1; *z* = 2.21; *p* = 0.026). Our data also show an increased engorgement rate in mosquitoes fed on infected blood versus uninfected blood (Table 3).

**Table 3:** Engorgement rate of *Anopheles darlingi* treated and untreated with antibiotics, and fed on *Plasmodium vivax* sample blood or uninfected blood.

Experimental group	Independent assays	Engorged/number of mosquitoes (%)	*p*-value
Treated Pv^+^	9	811/902 (89.9)	<0.0001
Untreated Pv^+^		726/900 (80.6)	
Treated Pv^−^	5	395/500 (79)	0.026
Untreated Pv^−^		364/500 (72.8)	

## Discussion

Some studies have used antibiotics to eliminate or reduce the bacterial microbiota in the anopheline midgut in order to enhance *Plasmodium* development and to study microbiota-parasite-vector interactions (Dong et al., 2009; Chouaia et al., 2012; Gendrin et al., 2015; Moreno et al., 2018). The importance of the role played by bacterial microbiota in mediating the immune response to *Plasmodium* infection in Asian and African malaria mosquitoes has been well documented (Beier et al., 1994; Dong et al., 2009). For example, the development of *P. falciparum*, *P. berghei*, *P. vinckei petteri* in *An. gambiae*, *An. stephensi* and *An. coluzzi* were enhanced when a reduction of bacterial load in the midgut was achieved using antibiotics before the ingestion of infected blood meals (Pumpuni et al., 1993; Dong et al., 2009; Sharma et al., 2013; Gendrin et al., 2015, 2016). To enhance *Plasmodium* development in anophelines, these previous studies either administered the antibiotic treatment for three days prior to blood-feeding or administered the antibiotics in the infected blood meal. However, information about the impact of antibiotic treatment on Neotropical vectors and on the development of *P. vivax* is scare. Moreno et al. (2018) assessed an antibiotic treatment (penicillin–streptomycin–20 μg/ml) administered with the blood meal to increase the infection of lab-reared *An. darlingi*; however, no effect on prevalence or infection intensity was observed. Consequently, in our experiments we administered the antibiotic treatment for three days prior to DMFA, as performed by Dong et al. (2009), and continued treatment the day after DMFA, treating infected mosquitoes daily until 14 dpi, as performed by Beier et al. (1994). Our aim was to test whether this approach would impact the susceptibility and survivorship of lab-reared *An. darlingi* infected with *P. vivax*.

First, our results show that antibiotic treatment over three days did impact the bacterial load of the cultivable midgut microbiota in mosquitoes, as previously demonstrated by Touré et al. (2000) and Dong et al. (2009). A reduction of bacterial load was also registered using qPCR to quantify the bacterial DNA copy numbers from midguts of antibiotic-treated mosquitoes. However, our results did not show strong evidence that continuous antibiotic treatment had a positive effect on prevalence in our lab-reared *An. darlingi*. In fact, our independent assays with lab-reared *An. darlingi* demonstrated high prevalence of infection in the untreated group (Supplementary Table S1), which may have interfered with the detection of any significant increase in prevalence resulting from antibiotic treatment, given that effects on prevalence are easier to detect in an experimental group when prevalence is low in the control (Churcher et al., 2012). In general, prevalence is higher in lab-reared *An. darlingi* while parasite intensity is low relative to Asian and African vectors (Moreno et al., 2018; Mohanty et al., 2021).

The effect of antibiotic treatment on parasite intensity was not statistically significant, however we observed lower levels of oocysts and sporozoites in treated mosquitoes, and a statistically significant decrease in sporozoite infection prevalence. Penicillin and streptomycin are commonly used in mosquito and parasite research without any known effect on mosquitoes or parasites (Sinden et al., 1985; Dong et al., 2009; Delves et al., 2012; Garver et al., 2012; Bando et al., 2013), while gametocyte production protocols indicate that gentamicin can negatively impact *P. falciparum* infection (Moll et al., 2013; Siciliano et al., 2020). However, Reader et al. (2015) observed that adding gentamicin to the culture medium as a preservative did not influence gametocyte production or viability, based on normal exflagellation and ATP production; and Beier et al. (1994) showed that exposing mosquitoes to a continuous treatment with gentamicin increased *P. falciparum* development in *An. gambiae* and *An. stephensi* in concentrations of 0.05% and 0.1%, respectively. Given that the effect of gentamicin on *P. vivax* development has yet to be studied and that continuous treatment with these three antibiotics did not increase *P. vivax* development in our study, it will be important for future studies to assess the effect of gentamicin on the *P. vivax* sporogonic cycle.

Recently Sharma et al. (2020) described a downregulation of antimicrobial peptide expression and a suppression of bacterial proliferation associated to *P. vivax* infection in *An. stephensi* model. It was previously hypothesized that *P. vivax* has the capacity to manipulate the detoxification system in the midgut of *An. aquasalis* (Bahia et al., 2013). The ability of *P. vivax* to serve its own development by manipulating the detoxification system of in the mosquito host may indirectly affect the bacteria present in the mosquito midgut (Bahia et al., 2013) by suppressing bacterial proliferation (Sharma et al., 2020). Since antibiotic treatment failed to increase the parasite load in the midgut and salivary glands in our study, the ability of *P. vivax* to manipulate its host and midgut microbiota needs to be further investigated in *An. darlingi* model.

The efficacy of the antibiotic treatment was confirmed in our study before blood feeding. Gendrin et al. (2015) observed that administering antibiotic in the blood meal reduced bacteria proliferation, but bacteria load increased when antibiotic was removed from a second blood feeding. In our experiment, the antibiotic treatment was continuous, except on the day of blood feeding, in order to maintain bacterial load at reduced levels until 14 dpi.

Besides *Plasmodium* development, mosquito survival plays an important role in malaria transmission (Reisen, 2017). Survival was increased by 10 days in *An. darlingi* females treated continuously with antibiotics, and increased survival was also noted in blood-engorged females treated with antibiotics. Thus, our observations demonstrate that antibiotic treatment does not reduce the lifespan of *An. darlingi* but may increase longevity. Furthermore, high mortality among infected mosquitoes decreased when mosquitoes were treated continuously with antibiotics. Increases in mosquito lifespan after antibiotic treatment have been observed in an *An. darlingi–P. vivax* model, under a concentration of 20 μg/ml of penicillin–streptomycin when given with blood meals (Moreno et al., 2018), and in an *An. gambiae–P. falciparum*/*P. berghei* model (Gendrin et al., 2015). In another study of *An. gambiae* infected with *P. falciparum*, mosquitoes were treated for only three days with 10 μg/ml-U/mL of penicillin–streptomycin and 15 μg/ml of gentamicin, and lower mortality was recorded in the control group until 7 dpi, despite a higher infection level (Dong et al., 2009). This is curious because the midgut microbiota play an important role in mediating the immune response in the midgut and in the formation of the peritrophic matrix, which is a physical barrier that envelops the blood meal to protect the midgut from pathogens in the digestion process (Hegedus et al., 2009; Song et al., 2018; Gao et al., 2020). However, the bacterial microbiota-mosquito interaction appeared to reduce mosquito survival regardless of infection or food source (blood or honey meal).

Other studies have suggested that the immune response that controls bacterial growth after blood feeding may also reduce mosquito fitness, and, consequently, increase mosquito mortality (Dong et al., 2009; Hughes et al., 2014; Gendrin et al., 2015). Furthermore, some species of bacteria may negatively affect mosquito survival, as observed in experimental co-infection with *P. vivax*, *P. falciparum* and *P. berghei* and *Serratia marcescens*, *Enterobacter amnigenus*, *Enterobacter cloacae*, *Klebsiella* spp. (Jadin, 1967; Seitz et al., 1987; Gonzalez-Ceron et al., 2003; Bahia et al., 2014). Therefore, antibiotic treatment may help control microbiota proliferation thereby positively affecting the mosquito fitness or lifespan (Hughes et al., 2014; Gendrin et al., 2015). Furthermore, antibiotic treatment did not negatively affect the engorgement rate of mosquitoes feeding on either infected or uninfected blood.

Ensuring mosquito survival to the sporozoite stage (~14 dpi) of *P. vivax* is important for sporozoite production. In the present study, and in our previous work (Santos et al., 2022), we have observed that mortality increases after 7 dpi. It is important to note that *P. vivax* infection increased the probability of death despite the antibiotic treatment. In fact, the probability of dying from infection was higher when looking at all 14 dpi. No difference was observed up to 7 dpi. Increased risk of mortality after 7 dpi may be associated with the release of sporozoites from the oocysts in the epithelium of the mosquito’s midgut. Sporozoite development in mosquitoes takes 9 days or longer, depending on temperature (Stratman-Thomas, 1940). In *An. darlingi*–*P. vivax* laboratory models, the incubation period lasts 9 days and takes ~14 days to reach maximum sporozoite load in the salivary glands (Moreno et al., 2018). Although, ruptured oocysts have already been observed at 7 dpi (data not shown).

Ferguson and Read (2002) suggested that mosquitoes may suffer greater physiological disturbances during the sporozoite stage than in the ookinete/oocyst stages of *Plasmodium* development. Furthermore, it is known that only around 19% of sporozoites released invade the salivary glands, and this sharp decline in the number of live sporozoites in the hemocoel may be due to hemocyte phagocytosis (Hillyer et al., 2007). In fact, in an *An. stephensi*–*P. vivax* model, Kumari et al. (2021) observed that the humoral immune response to free sporozoites in the hemocoel occurred in conjunction with a rapid proliferation of hemocytes. Previously, a high level of NO (nitric oxide) has been associated with sporozoite release of *P. berghei* in *An. stephensi* (Luckhart et al., 1998). Certainly, this interaction on sporozoite release may have strong effects on anopheline fitness and could explain the mortality we observed in *An. darlingi* infected by *P. vivax*.

In conclusion, continuous antibiotic treatment during *P. vivax* infection increased mosquito survival. However, antibiotic treatment did not have the dramatic impact on *P. vivax* development that has been observed for *P. falciparum* (Dong et al., 2009; Gendrin et al., 2015), and for rodent and avian malaria parasites (Gendrin et al., 2015; Martinez-de la Puente et al., 2021). These results call attention to the fact that *P. vivax*-Neotropical anophelines interaction remains under-investigated, especially with respect to tripartite microbiota-anopheline-*P. vivax* interaction. Additionally, given the fact that bacteria microbiota may negatively impact mosquito survival, future studies will need to investigate and compare the bacterial microbiota composition of untreated and antibiotic-treated mosquitoes. Immune gene expression study in *An. darlingi* mosquitoes along *P. vivax* development needs to be investigated in conjunction with further studies of bacterial load and composition in order to better understand microbiota-anopheline-*P. vivax* interaction and to elucidate the importance of bacterial microbiota in the *P. vivax* sporogonic cycle.

## Data availability statement

The original contributions presented in the study are included in the article/Supplementary material, further inquiries can be directed to the corresponding author.

## Ethics statement

The studies involving human participants were reviewed and approved by Ethics Committee at the Centro de Pesquisa em Medicina Tropical (CEPEM). The patients/participants provided their written informed consent to participate in this study.

## Author contributions

NS contributed to the study design, performed the experiments and the analyses, interpreted the results, and wrote the manuscript. FM and AA contributed to the experiments. AA and AB provided support with the mosquito colony. JM reviewed the manuscript. SP contributed to plasmid construction. MA designed and supervised the study, contributed to the interpretation of the results, and co-wrote the manuscript. All authors contributed to the article and approved the submitted version.

## Funding

This work was supported, in part, by the Brazilian Ministry of Health/DECIT/CNPq N° 23/2019 (grant number 442653/2019-0) and the Bill and Melinda Gates Foundation (INV-003970). Under the grant conditions of the Foundation, a Creative Commons Attribution 4.0 Generic License has already been assigned to the Author Accepted Manuscript version that might arise from this submission. The study also received funding from PPSUS/ Fundação de Amparo à Pesquisa do Estado de Rondônia (FAPERO) N° 001/2020 (grant number 0012.259726/2020-70), and the International Centers of Excellence for Malaria Research (ICEMR) program GR109237 (CON-80002357). NS expresses her appreciation for a fellowship granted by Coordenação de Aperfeiçoamento de Pessoal de Nível Superior–Brazil (grant number 88887.499061/2020-00).

## Conflict of interest

The authors declare that the research was conducted in the absence of any commercial or financial relationships that could be construed as a potential conflict of interest.

## Publisher’s note

All claims expressed in this article are solely those of the authors and do not necessarily represent those of their affiliated organizations, or those of the publisher, the editors and the reviewers. Any product that may be evaluated in this article, or claim that may be made by its manufacturer, is not guaranteed or endorsed by the publisher.
