# Supplementary material for: Assessment of antibiotic treatment on Anopheles darlingi survival and susceptibility to Plasmodium vivax
Source: Front Microbiol. 2022 Oct 5;13:971083. doi: 10.3389/fmicb.2022.971083 (PMC9583876; doi:10.3389/fmicb.2022.971083)
Supplement: Supplementary file 1 [file Data_Sheet_1.docx]

Supplementary Material


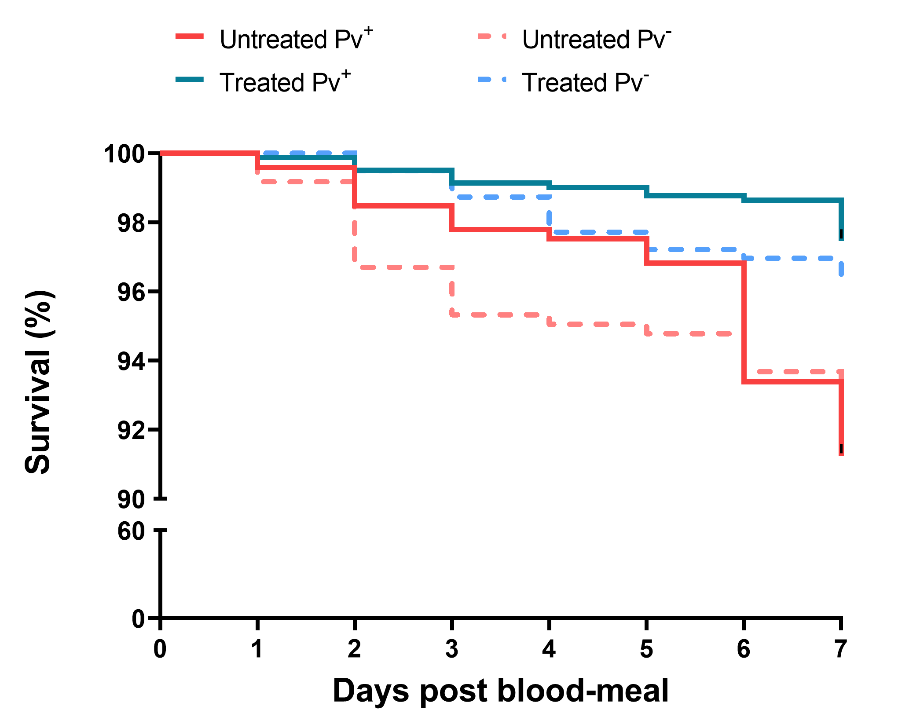


**Supplementary Figure S1.** Survival curves until 7 dpb for mosquitoes treated and untreated with antibiotic and fed on *P. vivax* positive samples or on uninfected blood samples.

|  | **Reference in the model** | | | |
| --- | --- | --- | --- | --- |
|  | **Untreated Pv^+^** | **Untreated Pv^-^** | **Treated Pv^-^** | **Treated Pv^+^** |
| **Untreated Pv^+^** | - | 1.27 | 2.44* | 3.02* |
| **Untreated Pv^-^** | **0.78** | - | 1.91 | 2.37* |
| **Treated Pv^-^** | 0.41* | 0.52 | - | **1.23** |
| **Treated Pv^+^** | 0.33* | 0.42* | 0.80 | - |
|  |  |  |  |  |

**Supplementary Table S2.** Hazard ratio estimated by Cox model until 7 days post blood-meal. Bold number highlights the impact of infection estimated by the model.
